# A pH-Responsive Supramolecular Drug Delivery System Constructed by Cationic Pillar[5]arene for Enhancing Antitumor Activity

**DOI:** 10.3389/fchem.2021.661143

**Published:** 2021-04-12

**Authors:** Luzhi Liu, Qingqing Zhou, Qin He, Wengui Duan, Yan Huang

**Affiliations:** ^1^School of Chemistry and Chemical Engineering, Guangxi University, Nanning, China; ^2^Guangxi Key Laboratory of Electrochemical Energy Materials, Nanning, China; ^3^Guangxi Institute of Chinese Traditional Medical & Pharmaceutical Science and Guangxi Key Laboratory of Traditional Chinese Medicine Quality Standards, Nanning, China

**Keywords:** pillar[5]arene, supramolecular vesicles, pH-responsive, drug delivery systems, anti-tumor activities

## Abstract

Drug delivery systems have good biocompatibiliy and low side effects for cancer treatment, but overcoming high efficiency of drug-loading and the drug-targeting controlled release still remains challenging. In this work, supramolecular vesicles, with pH-triggering effect, have been successfully constructed for drug delivery, which are fabricated by the complexation between a cationic pillar[5]arene (DAWP5) and a sodium dodecyl sulfonate (SDS) in aqueous solution. Drug-loading and releasing results demonstrated that anticancer drug doxorubicin (DOX) could be loaded efficiently by such cationic vesicles in neutral condition, and the drug release could be controlled in the simulated weak acid environment of tumor cells. Moreover, the vesicles had low cytotoxicity to normal human cell (L02), while the DOX-loaded vesicles could significantly enhance the cytotoxicity of free DOX for normal cell L02 and four tested tumor cells (Hela, HepG2, MGC-803 and T24). Especially for HepG2, after 24 h incubation time, IC_50_ of DOX-loaded vesicles was only 0.79 μM, about 23% of that of DOX (3.43 μM). These results suggested that such novel vesicles have promising potential to construct nano-drug delivery systems for various biomedical applications.

## Introduction

Drug delivery systems (DDSs) constructed by nano carriers have attracted tremendous attention due to their better biocompatibiliy and lower side effects over the traditional naked drugs, which are considered to be one of the most active and promising treatment for cancer in future clinical applications (Zhou et al., 2017a; Rawal and Patel, 2019; Yang et al., 2019; Zhang et al., 2019a). Therefore, designing smart drug delivery systems with high drug loading efficiency and drug controlled-release in tumor microenvironment have been a hot topic (Gao et al., 2019; Xiao et al., 2019). Up to now, various nanoparticles, especially vesicles (Massiot et al., 2019; Elsharkasy et al., 2020; Skotland et al., 2020) constructed by building blocks have been widely used in drug delivery due to their excellent drug loading performance and adjustable stimulus-response ability. However, the fabrication of drug delivery systems require precise design and complicated synthesis of building blocks, most of which are polymer amphiphiles (Cui et al., 2019; Ulrich, 2019; Zhang et al., 2019b). Among the nano-drug-loaded carriers, supramolecular vesicles (SVs) based on non-covalent interactions, on the one hand, can avoid tedious multistep synthesis and purification. On the other hand, they can show reversible self-assembly and tunable functionailzation, which make them ideal candidates for drug delivery.

pillar[n]arenes, as a new class of macrocycle first synthesized by Ogoshi et al. (2008), have recently been widely used in many fields including molecular recognition (Yakimova et al., 2016; Yuan et al., 2017), mechanical interlocking molecules (Chen et al., 2013a; Zhang et al., 2019c), artificial transmembrane channels (Chen et al., 2013b; Xin et al., 2017), metal ion separation (Xia et al., 2017), chemical sensors (Wu et al., 2014; Stepanova et al., 2017; Wei et al., 2017; Hua et al., 2018; Sun et al., 2018), and supramolecular polymers (Strutt et al., 2012; Zhou et al., 2014a; Nierengarten et al., 2016; Zhang et al., 2018). At present, researchers are more focusing on the study of water-soluble pillar[n]arenes (Hu et al., 2016; Yang et al., 2016; Yu et al., 2016; Zhou et al., 2017b; Li et al., 2019; Zhong et al., 2019) because they can effectively bind different types of molecules into their cavities to construct a series of stimulus-responsive supramolecular amphiphiles for the application in DDSs. Among the DDSs, the pillararenes, as building units, are mostly based on the design of negative ion or neutral amphiphilic. In order to better control the drug targeted release and achieve the efficient therapy, complex construction methods are usually adopted for pillararene-based self-assembly, such as using gold nanoparticles (Ahn et al., 2020), metal–organic frameworks (Wu et al., 2018a), polymers (Santos et al., 2020) etc. as assembly platforms. However, sometimes the gains are contradictory, such as the report (Wu et al., 2018b) that the nanoparticels PUWPFa NPs can achieve efficient synergistic chemophotothermal therapy, but long time for the drug release. In addition, even in the simple host-guest system, most of the designed guest molecules are pyridine compounds with certain toxicity. By comparison, positively charged pillararenes, as mediators between nucleic acids and anionic lipidsin gene therapy, can be used to fabricate DDSs for drug/siRNA co-delivery. Pei first reported a redox responsive cationic vesicle self-assembled by ferrocenium capped amphiphilic pillar[5]aren for drug/siRNA co-delivery (Chang et al., 2014). The DDSs can not only enhance the bioavailability of drugs to cancer cells, reduce the adverse side effects to normal cells, but also overcome the drug resistance of cancer cells, indicating that cationic vesicles have unique advantages in drug delivery. Moreover, caition pillar[n]arene can also be assembled into nanoparticles with non/low toxic anionic guest such as ATP for drug delivery (Zhou et al., 2014b). However, there are few reports about the cationic nano carriers assembled by amphiphilic pillararenes for drug delivery. Therefore, the construction of cationic drug-loaded carriers are not only of great significance for the search of nanomedicines with excellent anticancer activity, but also have good complementarity with the anionic carriers formed by pillararene.

**Figure 1:**
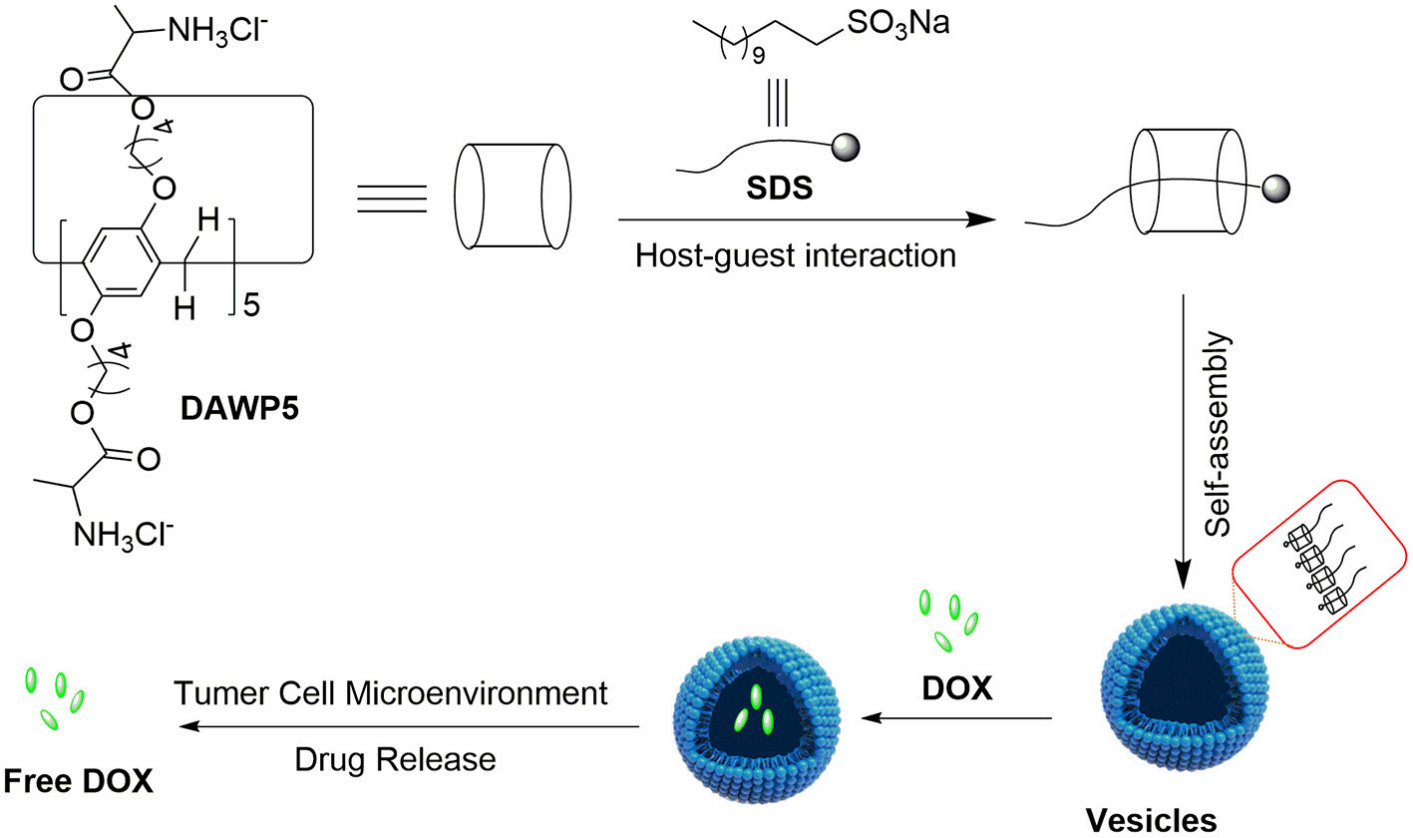
Illustration of constructing supramolecular vesicles and the application of supramolecular vesicles in anticancer drug delivery.

Herein, we have designed a D-alanine-modified water-soluble pillar[5]-arene (DAWP5). The DAWP5 could interact with sodium dodecyl sulfonate (SDS) to form a inclusion complex in aqueous solution, and then further self-assemble into pH-sensitive cationic vesicles SDS⊂DAWP5 (Figure 1). Their application in drug delivery *in vitro* was investigated. The results showed that the vesicles could efficiently load anticancer drug DOX, and control the drug release in the simulated weak acid environment of tumor. Furthermore, the vesicles had low cytotoxicity to normal human cell, while the drug-loaded system DOX⊂SDS⊂DAWP5 could markedly enhance the cytotoxicity of DOX against the normal cell L02 and four kinds of tumor cells (Hela, HepG2, MGC-803, and T24), which may have a promising application in cancer treatment.

## Materials and Methods

### Reagents and Instruments

The D-alanine-modified water-soluble pillar[5]arene DAWP5 was produced in our laboratory as described (Supplementary Scheme 1). Other reagents were purchased from commercial suppliers and used as received. The NMR spectra were recorded on a Bruker Avance III HD 600 MHz spectrometer (Bruker Co., Ltd., Zurich, Switzerland), and the chemical shifts are expressed in ppm (δ) downfield relative to TMS, as an internal standard. The UV–vis absorption spectra were performed on a Shimadzu UV-1800 spectrometer (Shimadzu Corp., Kyoto, Japan). Transmission electron microscope (TEM) investigations were carried out on a Tecnai G2 F20 S-TWIN instrument. Zeta-potential measurements were performed on a Zetasizer Nano S (Malvern instruments Ltd, UK). Atomic force microscopy (AFM) investigations were carried out on a Bruker Dension Icon (Bruker Co., Ltd., Zurich, Switzerland). Dynamic light scattering (DLS) measurements were carried out on a Mastersizer 2000 system (Malvern instruments Ltd, UK).

### Preparation of the DOX-Loaded Vesicles

DOX-loaded vesicles were fabricated as follows: To a mixture aqueous solution of DAWP5 and SDS, a certain amount of DOX was added. And the concentrations of DOX, DAWP5, and SDS were adjusted to 0.33 mM, 0.33 mM and 1 mM, respectively. After sonicating 30 min and then standing overnight, the DOX-loaded vesicles were prepared and purified by dialyzing in distilled water (molecular weight cutoff 3500), until the water outside the dialysis showed negligible DOX absorption. The DOX encapsulation efficiency (EE) and loading efficiency (LE) were calculated by the following equation.

(1) $$\begin{array}{r} \text{EE}\left(\text{\%}\right)=\left({\text{m}}_{\text{DOX}-\text{loaded}}/{\text{m}}_{\text{DOX}}\right)\times 100 \end{array}$$

(2) $$\begin{array}{r} \text{LE}\left(\text{\%}\right)=\left({\text{m}}_{\text{DOX}-\text{loaded}}/{\text{m}}_{\text{NPs}}\right)\times 100 \end{array}$$

Here m_DOX−loaded_, m_DOX_ and m_NPs_ are mass of DOX encapsulated in vesicles, DOX added, and DOX-loaded vesicles, respectively. The amount of unloaded DOX in the dialysate was determined by UV-vis at 490 nm.

### pH-Responsive Behavior of the DOX-Loaded Vesicles

Aqueous solution (pH = 7.0) and disodium hydrogen phosphate citric acid buffer (pH = 6.2 and 4.8) were used as drug release media to simulate normal physiological environment and acidic tumoral environment. Five milliliter of DOX-loaded vesicles in a dialysis bag was added into 15 milliliter of appropriate release medium at 37°C. At specified time intervals, 2 milliliter of the release media was taken out for measuring the concentrations of released DOX by UV-vis absorption at 480 nm and then was returned to the original release media.

### *In vitro* Cell Assay

HeLa (Human cervical cancer) cells, HepG2 (Human hepatoma) cells, MGC-803 (Human gastric cancer) cells, T24 (Human bladder cancer) cells and human normal liver cells L02 were cultured in DMEM medium containing 10% fetal bovine serum and 1% penicillin/streptomycin in 5% CO_2_ at 37°C. MTT assay was used to evaluate the relative cytotoxicity of vesicles, DOX and DOX-loaded vesicles. Four kinds of tumor cells were seeded in 96 well plates and grew at 37°C for 24 h. Then they were incubated with different concentrations of unloaded vesicles, free DOX, and DOX-loaded vesicles for 6, 12, 24, and 48 h. Subsequently, the cells were washed and further incubated for 4 h with fresh culture medium containing MTT. After removing the MTT medium, DMSO was added into the well, and the plates were carefully shaken until the formazan crystals were fully dissolved. Finally, the absorbance (OD) of each well was measured by enzyme-linked immunosorbent assay (ELISA). All experiments were carried out with four replicates.

## Results and Discussion

### Size, Morphology, and pH-Responsive of DAWP5-Based Assemblies

**Figure 2:**
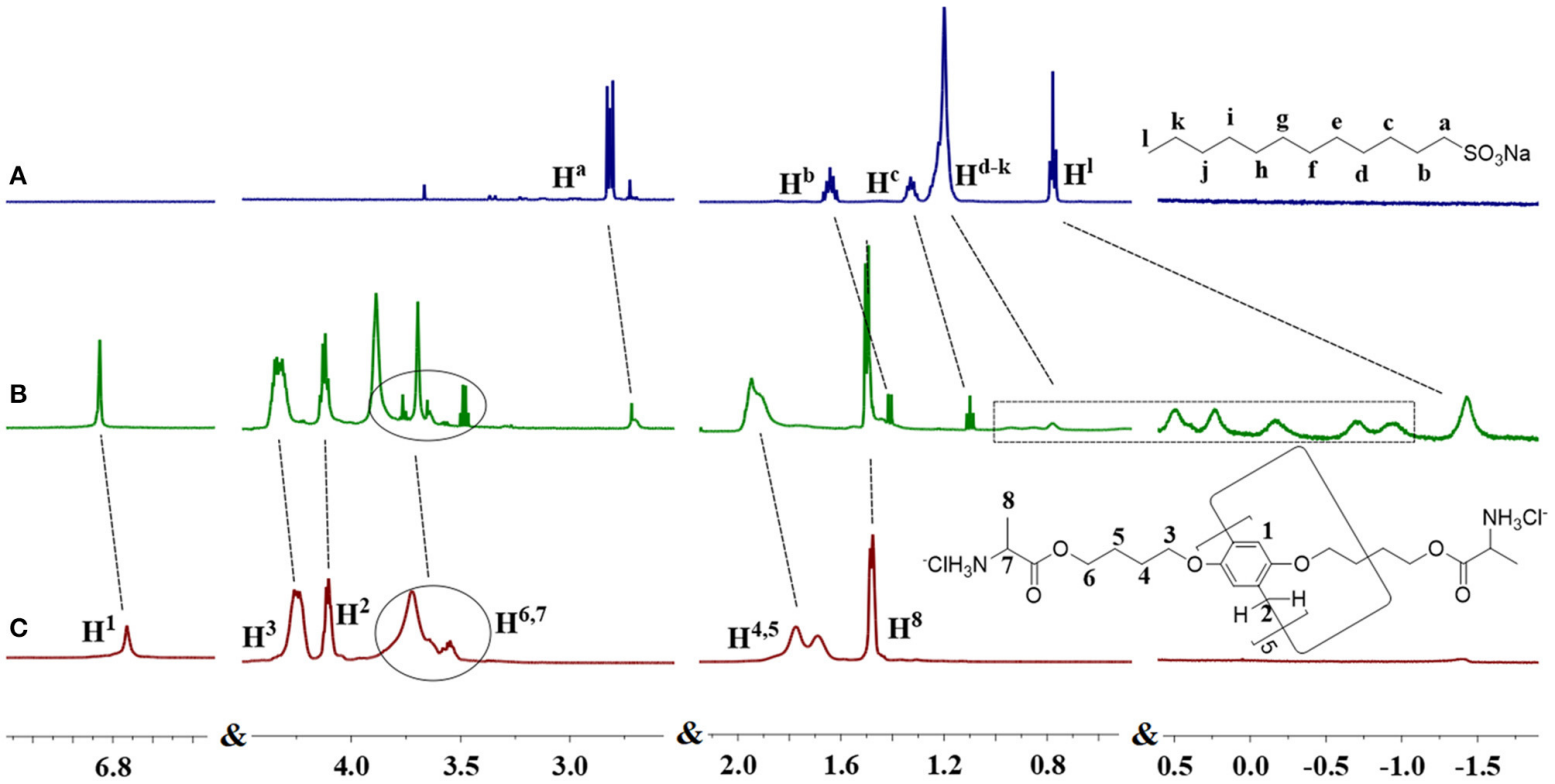
^1^HNMR spectra (600MHz, D_2_O, 298K): **(A)** SDS, **(B)** SDS+1eq. DAWP5, and **(C)** DAWP5.

Initially, the host-guest complexation between DAWP5 and guest SDS was studied by ^1^HNMR measurements, as shown in Figure 2. After addition of DAWP5 (1 equiv) to the SDS solution in D_2_O, all the protons in the alkyl chain of SDS shifted to high field in varying degrees. Especially for H^d−i^, their signals showed remarkable upfield shifts and accompanied with line broadening due to the protons shielded in the inner electron-rich cavity of DAWP5. Meanwhile, the protons of H^1^, H^3^, and H^4, 5^ of DAWP5 slightly moved to downfield in presence of SDS. Therefore, the SDS could thread into the cavity of DAWP5 to form a host-guest complex, which might be mainly driven by the cooperative CH-π, electrostatic and hydrophobic interaction (Sun et al., 2019; Zhu et al., 2019). Subsequently, the stoichiometry of complexation between DAWP5 and SDS was studied by Job plot method (i.e. UV titration experiments), and the binding stoichiometry of the inclusion host-guest complex SDS@DAWP5 was 1:1 (Supplementary Figure 10). After affirming the binding stoichiometry, the association constant (Ka) of SDS@DAWP5 was calculated to be (3.5 ± 0.1) × 10^3^ M^−1^.

Cationic water-soluble pillar[5]arene DAWP5 is an amphiphilic molecule and has a high binding constant with SDS, thus the host-guest system can be utilized to construct the pH-sensitive supramolecular assemblies. By subjecting a aqueous solution of DAWP5 and SDS and sonicating for 30 min, a distinct Tyndall effect could be observed (Supplementary Figure 11), indicating that the supramolecular assemblies were formed by their host-guest interaction. The best molar ratio between DAWP5 and SDS for constructing such assemblies was investigated by UV-vis titration experiments, as shown in Supplementary Figure 12. With the increase of concentration of SDS, the absorption intensity of DAWP5 at 294 nm enhanced gradually and reached a maximum value. When the SDS/DAWP5 ratio was 3:1, the absorbance tended to balance. It was proved that this point ([SDS]:[DAWP5]=3:1) was the best molar ratio for constructing the supramolecular assemblies between DAWP5 and SDS.

**Figure 3:**
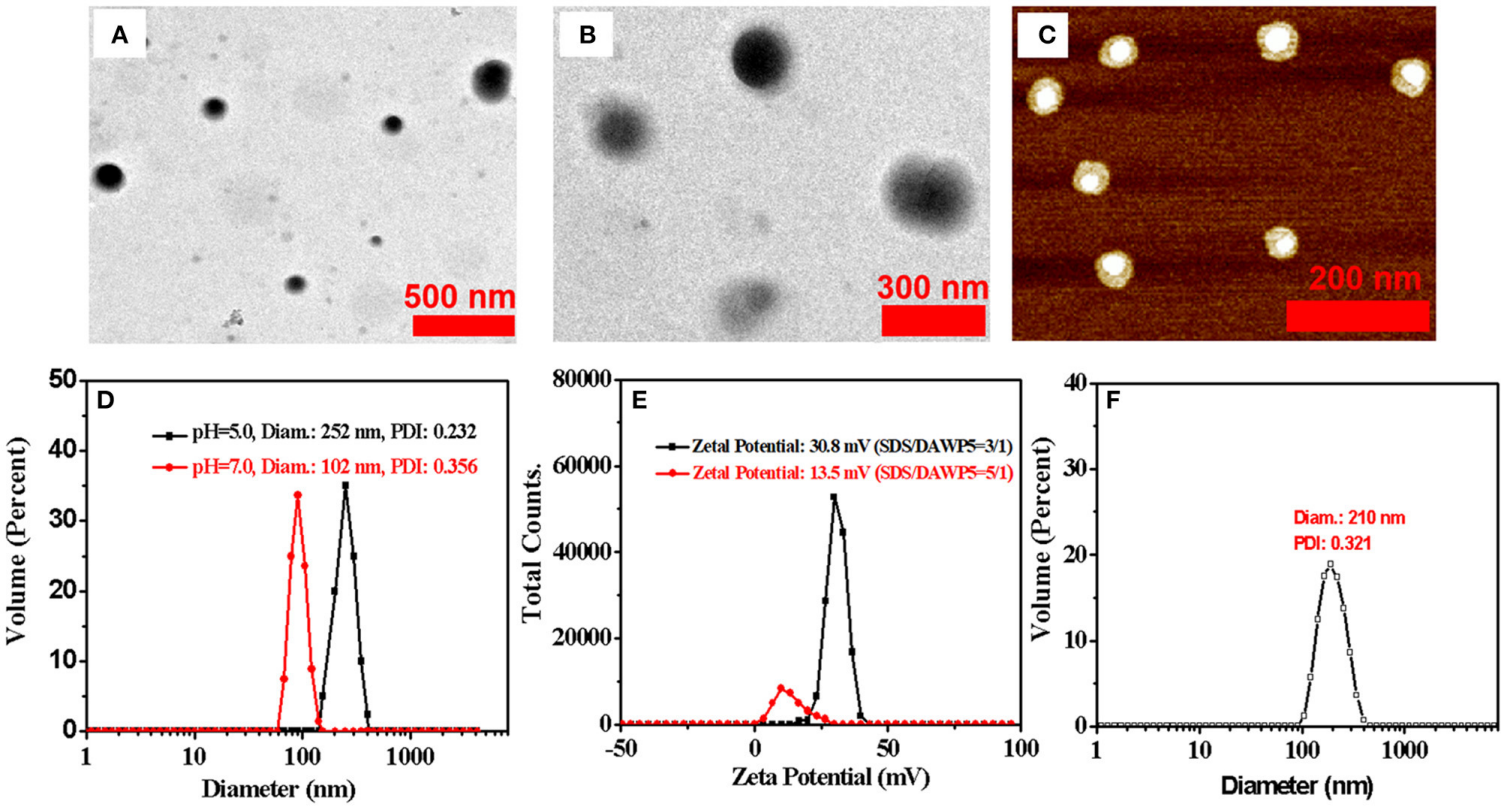
**(A)** TEM images of SDS⊂DAWP5 (3/1) vesicles. **(B)** TEM images of DOX⊂SDS⊂DAWP5 (3/1) vesicles. **(C)** AFM images of SDS⊂DAWP5 (3/1) vesicles. **(D)** DLS data of SDS⊂DAWP5 vesicles in different pH condition. **(E)** zeta potential results of SDS⊂DAWP5 vesicles in different molar ratios of SDS:DAWP5 and **(F)** DLS data of DOX⊂SDS⊂DAWP5 vesicles.

Next, the morphology and size of supramolecular assemblies were monitored by transmission electron microscopy (TEM), dynamic light scattering (DLS), and atomic force microscopy (AFM) (Figure 3). The TEM results confirmed that the spherical components were actually vesicles with an average diameter of 100 nm, which were consistent with the results determined by DLS (Figure 3C). Meanwhile, the morphology and wall thickness of the vesicles were further confirmed by AFM (Supplementary Figure 13). The height of the vesicle, i.e., the thickness of the two walls of the vesicle, was 3.32 nm, suggesting the thickness of the vesicle was 1.66 nm, which was close to the length of a SDS guest (1.67 nm calculated with the energy-minimized structure). It turned out that the vesicles were formed by SDS@DAWP5 in a mono-layer packing mode. In addition, the vesicles SDS⊂DAWP5 formed at the mole fraction of 3:1 (SDS/DAWP5) had large positive zeta potential (30.8 mV), which indicated the vesicles had strong repulsive force, basically due to excessive ammonium salt cation of host. Therefore, the vesicles formed at the best ratio between DAWP5 and SDS were suitable for stable existence in aqueous solution.

Subsequently, the pH-responsive behavior of the supramolecular vesicles was investigated. When the nanoparticles were dispersed in buffer solution and the pH was adjusted to 5.0, the Tyndall effect weakened clearly (Supplementary Figure 11) and the size of the assemblies in DLS increased significantly (Figure 3D and Supplementary Figure 14). The main reason is that after adjusting the pH value, the supramolecular self-assembly had partially dis-assembled, resulting in the decrease of the number of nanoparticles in the system. At the same time, some nanoparticles would be reorganized into larger assemblies. Thus, such supramolecular assemblies had good pH-responsiveness.

Furthermore, we investigated the influence of the mole ratio between host and guest on the morphology and size of vesicles. When the supramolecular assemblies formed at the mole fraction of 5:1 (SDS/DAWP5), the diameter of vesicles was ca. 220 nm in the SEM experiments, which was close to the average hydrated particle size of 250 nm as shown by DLS (Supplementary Figures 14, 15). These results clearly showed that the size of the vesicles was dependent on the molar ratio of SDS/DAWP5. When the molar ratio of SDS/DAWP5 increased from 3/1 to 5/1, the size of vesicles became larger and increased from 100 nm to ca. 250 nm. Moreover, the zeta potential of vesicles dropped sharply to 13.5 mV (Figure 3E), indicating that it was less stable for this kind of vesicles than that assembled in the 3/1 molar ratio of SDS/DAWP5. Considering that the repulsion induced stability of nanoparticles increased, and small nanoparticles were beneficial to the treatment of refractory tumors (Cabral et al., 2011), the molar ratio of [SDS]/[DAWP5] = 3:1 was selected to obtain more stable and smaller vesicles, and their drug loading behavior and application in drug delivery were further studied.

### Drug Controlled Release of DAWP5-Based Assemblies

**Figure 4:**
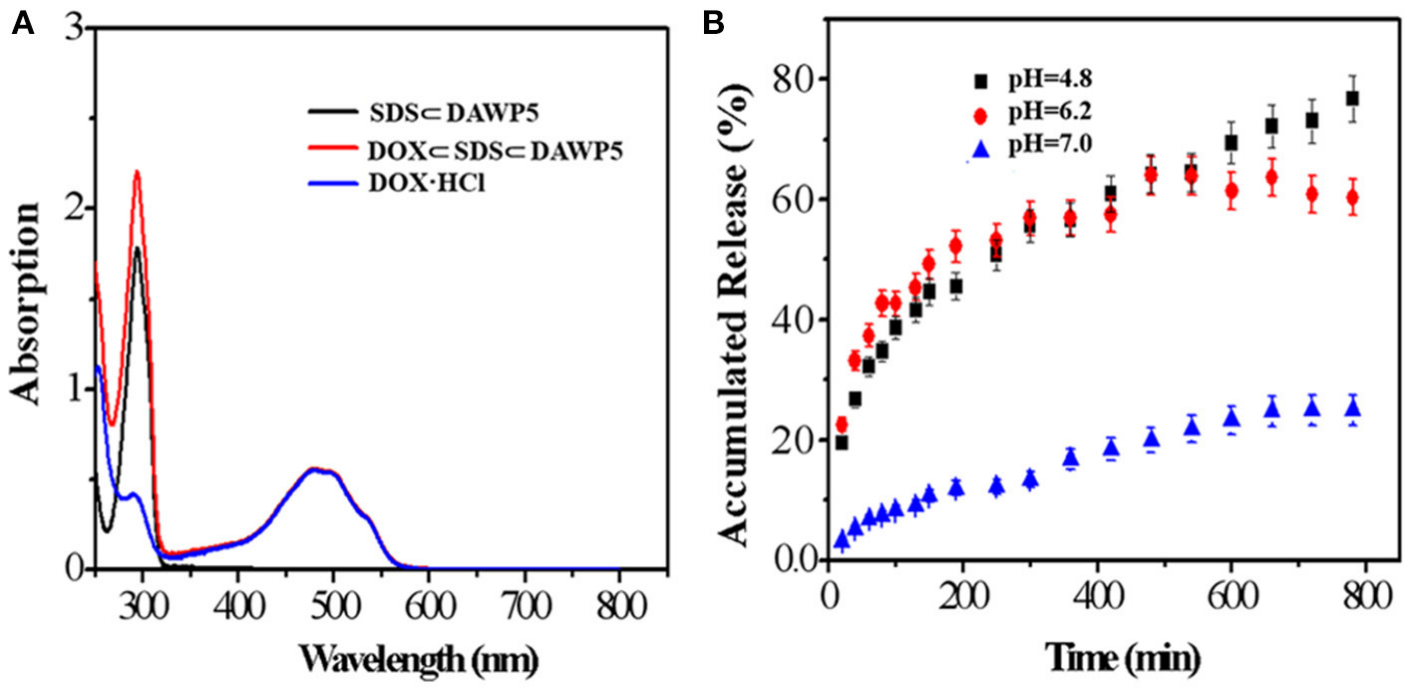
**(A)** UV-vis spectra of SDS⊂DAWP5 vesicles, DOX and DOX⊂SDS⊂DAWP5 vesicles. **(B)** Time-dependent drug release of DOX⊂SDS⊂DAWP5 vesicles under different pH conditions.

To study the drug release behavior and encapsulation efficiency of the pH-responsive cation vesicles, doxorubicin (DOX), a hydrophobic anticancer molecule, was selected as the model compound. In order to prepare DOX-loaded vesicles, the aqueous solution of drug DOX was added quickly to the aqueous solution containing DAWP5 and SDS ([SDS]/[WP5] = 3:1). After interaction and purification by dialysis, the UV/Vis absorption of the DOX-loaded vesicles was strong in the range of 400–700 nm, which featured the characteristic absorption of DOX in aqueous solution (Figure 4A). Furthermore, TEM and DLS experiments showed that the size of the DOX-loaded vesicles became much larger (ca. 210 nm, Figure 3) than those of the unloaded vesicles (100 nm), which confirmed that the DOX drugs were successfully loaded into the vesicle cavities. The DOX encapsulation and loading efficiency were calculated to be 75.1 and 10.0%, respectively, by the UV-absorption spectra.

The release behavior of drug-loaded vesicles was examined under acidic pH environment. As shown in Figure 4B, when the pH value of drug-loaded system was adjusted to 4.8, by simulating the endolysosomal environment, the amount of DOX release from the DOX-loaded vesicles reached a maximum of 72.2% within 13 h. The main reason for the drug-sustained release in a simulated tumor microenviroment was that the cationic vesicles would undergo partial disassembly or reorganization at lower pH. In comparison, the DOX-loaded vesicles in neutral conditions (pH = 7.0) remained relatively stable and 37.0% of DOX was released in the same time. The behavior for long-term drug-release under pH stimulation was beneficial to improve the therapeutic effect of drug and reduced the side effects, especially for drug with short half-life. Combining with the slightly acidic environment of tumor cell, such DOX loaded vesicle could be used to trigger tumor tissue, which was an ideal candidate for DDSs.

### Cytotoxicity of DAWP5-Based Assemblies

Cytotoxicity of unloaded and DOX-loaded vesicles to normal cell L02 and four tumor cells including HeLa, HepG2, MGC-803 and T24 cells were evaluated by the methyl thiazole tetrazolium (MTT) assay. As shown in Table 1, the results showed that the unloaded vesicles had relatively low cytotoxicity (IC_50_ > 10 μM) to the normal cell and four kinds of tumor cells after the incubation between the vesicles and cells for 48 h, indicating that the cationic vesicles constructed from pillar[5]arene modified by alanine units had low cytotoxicity, which was great significant for the application in DDSs. However, after loading the drug DOX, the vesicles clearly enhanced the cytotoxicity of DOX aganist both the normal cell L02 and tumor cells. The main reason maybe that the drug delivery systems increased cells uptake efficiency.

**Table 1:** Inhibitor activities of vesicles SDSÌDAWP5, dox-loaded vesicles DOXÌSDSÌDAWP5, and free DOX·HCl against to four tumor cells and normal cell.

**Cells**	**IC50 (*******μ*******M)**							
	**DOX⊂SDS⊂DAWP5**				**DOX·HCl**				**SDS⊂DAWP5**
	**6 h**	**12 h**	**24 h**	**48 h**	**6 h**	**12 h**	**24 h**	**48 h**	**48 h**
Hela	>10	1.21 ± 0.20	0.65 ± 0.11	0.44 ± 0.06	>10	2.5 ± 0.17	0.78 ± 0.06	0.71 ± 0.09	>10
HepG2	>10	1.94 ± 0.23	0.79 ± 0.09	0.40 ± 0.08	>10	4.45 ± 0.34	3.43 ± 0.06	0.70 ± 0.07	>10
MGC-803	>10	4.38 ± 0.54	1.67 ± 0.04	0.49 ± 0.06	>10	4.64 ± 0.47	2.40 ± 0.22	0.94 ± 0.06	>10
T-24	>10	2.72 ± 0.03	1.09 ± 0.07	0.68 ± 0.01	>10	2.79 ± 0.03	1.76 ± 0.45	1.35 ± 0.08	>10
L-02	–	–	–	1.21 ± 0.14	–	–	–	4.94 ± 0.72	>10

**Figure 5:**
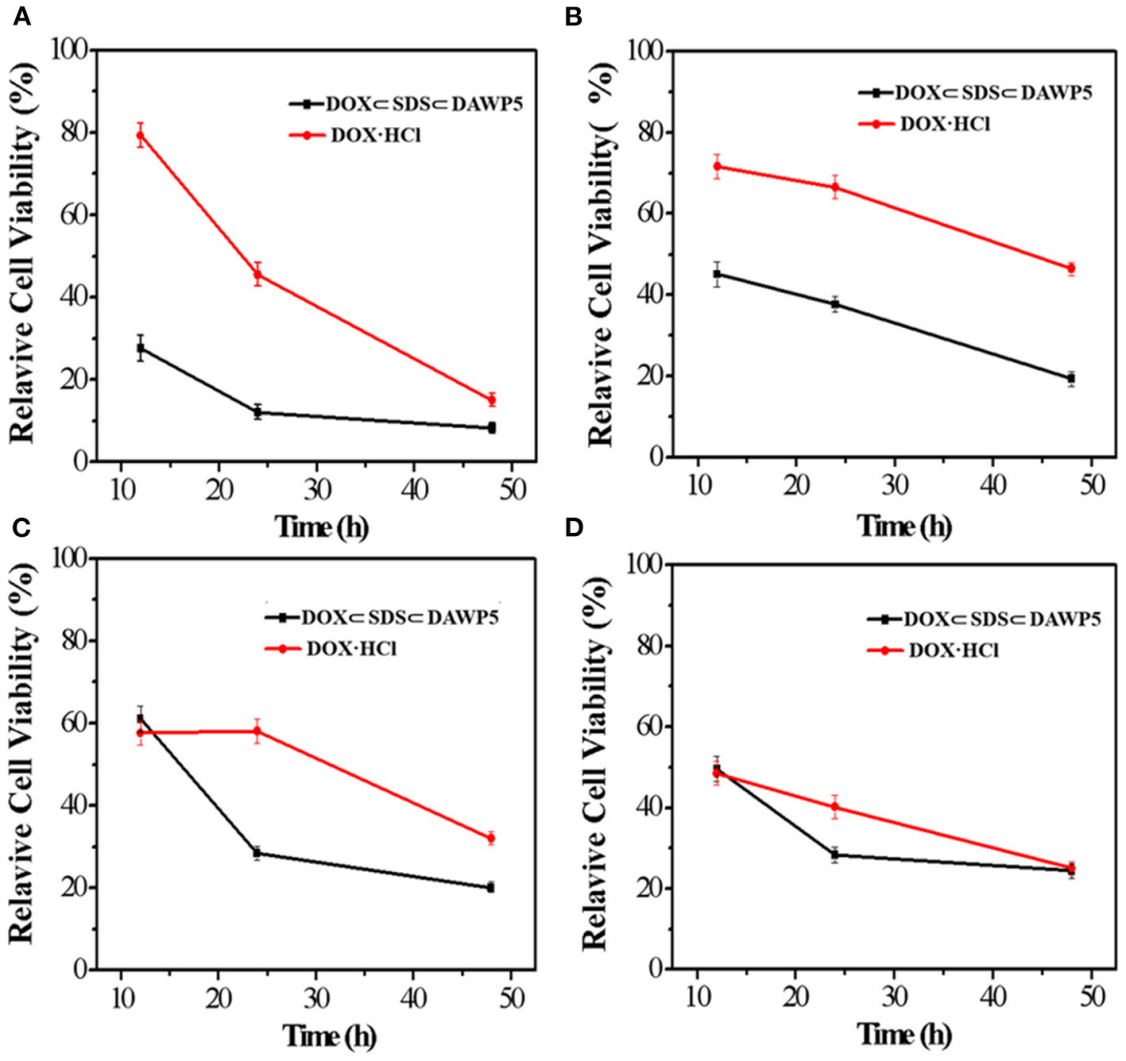
*In vitro* cytotoxicities of DOX-loaded vesicles and free DOX against **(A)** HeLa, **(B)** HepG2, **(C)** MGC-803, and **(D)** T24.

In order to evaluate the anticancer efficiency of DOX-loaded vesicles, HeLa, HepG2, MGC-803, and T24 cells were incubated with DOX-loaded vesicles and free DOX for 12, 24 and 48 h, respectively. As shown in the Figure 5, the results showed that DOX-loaded vesicles exhibited markedly stronger inhibitory activity against all the tested tumor cells than free DOX at the three tested time periods. Moreover, The DOX-loaded vesicles displayed excellent anticancer efficiency after incubation with cancer cells within a short time. However, free DOX with the same anticancer efficiency required longer incubation time with cells. Especially for HepG2 and T24 cells, after 24 h of incubation with drug, their IC_50_ of DOX-loaded vesicles were 0.77 μM and 1.09 μM, which were 23 and 62% of free DOX, respectively (IC_50_ of free DOX for HepG2 and T24 were 3.43 μM and 1.76 μM, respectively). To our knowledge, the enhancement effect on the cytotoxicity of these tumor cells was the highest among the DOX-loaded pillararene-based assemblies. It should be noted that the inhibitory activity of the DOX-loaded vesicles on these two kinds of cells in 24 h incubation time was close to or even higher than that of free DOX in 48 h. Therefore, such supramolecular vesicles have great potential in drug delivery or (and) cancer therapy.

## Conclusions

In summary, a novel type of cationic vesicles with low cytotoxicity based on host-guest complexation between DAWP5 and SDS, have been developed for pH-triggered drug delivery. Drug-loading and releasing *in vitro* results demonstrated that DOX can be encapsulated successfully and efficiently by cationic vesicles in deionized water, and sustain-released within 13 h in the simulated weak acid environment of tumor cells. Furthermore, MTT assay was used to evaluate the cytotoxicity of the DOX-loaded vesicles, and the results showed that such drug-loaded nanoparticles exhibited markedly stronger inhibitory activity against both normal cell and four kinds of tumor cells than free DOX. More importantly, compared with free DOX, the DOX-loaded vesicles displayed strong cytotoxicity in a shorter incubation time with tumor cells. Therefore, such DOX-loaded vesicles may be expected to develop novel nano-anticancer drug with potential applications.

## Data Availability Statement

The original contributions presented in the study are included in the article/Supplementary Material, further inquiries can be directed to the corresponding authors.

## Author Contributions

All authors listed have made a substantial, direct and intellectual contribution to the work, and approved it for publication.

## Conflict of Interest

The authors declare that the research was conducted in the absence of any commercial or financial relationships that could be construed as a potential conflict of interest.
