# Supplementary material for: A pH-Responsive Supramolecular Drug Delivery System Constructed by Cationic Pillar[5]arene for Enhancing Antitumor Activity
Source: Front Chem. 2021 Apr 12;9:661143. doi: 10.3389/fchem.2021.661143 (PMC8072374; doi:10.3389/fchem.2021.661143)
Supplement: Supplementary file 1 [file Data_Sheet_1.pdf]

A pH-Responsive Supramolecular Drug Delivery System Constructed by Cationic Pillar[5]arene for Enhancing Antitumor Activity

Luzhi Liu[1,3*], Qingqing Zhou[1], Qin He[1], Wengui Duan[1*], Yan Huang[2*]

[1]School of Chemistry and Chemical Engineering, Guangxi University, Nanning 530004, Guangxi, P. R. China

[2]Guangxi Institute of Chinese Traditional Medical & Pharmaceutical Science and Guangxi Key Laboratory of Tradtitional Chinese Medicine Quality Standards, Nanning 530022, Guangxi, P. R. China

[3]Guangxi Key Laboratory of Electrochemical Energy Materials, Nanning, Guangxi 530004, P. R. China

*** Correspondence:**
Luzhi Liu
llzh068@163.com
Wengui Duan
wgduan@gxu.edu.cn
Yan Huang
hy2002-2006@163.com

Supplementary Material

1 Synthesis of water soluble pillar[5]arene DAWP5..2

2 Job plot of complex SDS@DAWP5 and its association constant (Ka)........................10

3 Tyndall Effects of DAWP5 and SDS⊂ DAWP5 Vesicles in Different pH........................11

4 Determination of the Best Molar Ratio of DAWP5 and SDS for Aggregation........................11

5 The Wall Thickness of Vesicles SDS⊂ DAWP5 and the Length of SDS........................12

6 DLS data of SDS⊂ DAWP5 Vesicles (SDS/ADWP5=5/1) in Different pH........................12

7 SEM of SDS⊂ DAWP5 Vesicles (SDS/ADWP5=5/1)........................13

1 Synthesis of water soluble pillar[5]arene DAWP5

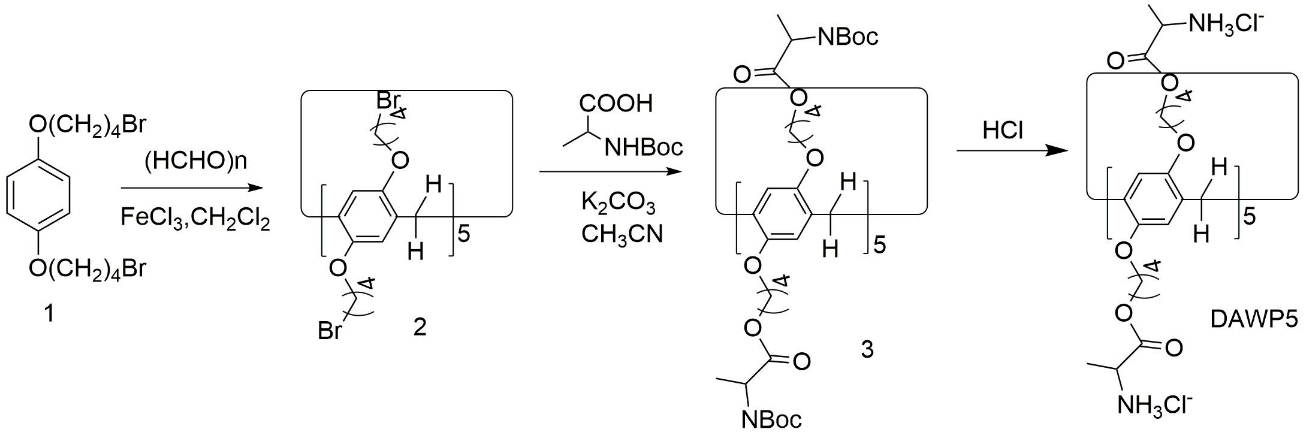

SCHEME S1 Synthesis route of water soluble pillar[5]arene **DAWP5**

Compound ***2***

To a solution of 1 (4.2 g, 11.0 mmol) in CH_2Cl_2 (500 mL), paraformaldehyde (0.99 g, 33 mmol) was added in a nitrogen atmosphere. Then, anhydrous ferric chloride (0.285 g, 1.76 mmol) was added to the solution and the mixture was stirred at 30 °C. After the completion of the reaction, water (100 mL) was added and the product was extracted with CH_2Cl_2 (3 ×40mL). The combined organic phase was dried over with anhydrous Na_2SO_4. The crude mixture was chromatographed over silica gel column using a mixture of ethyl acetate and petroleum ether giving compound **2** as white solid. Yield 55%, mp 90.4-91.3 °C; ^1H NMR (600 MHz, $CDCl_3$, 298K) δ/ppm: 6.82 (s, 10H), 3.93 (s, 20H), 3.75 (s, 20H), 3.44 (s, 20H), 2.06 (s, 20H), 1.93 (s,20H); ^{13}C NMR (151 MHz, $CDCl_3$, 298K) *δ*/ppm; 149.8, 128.3, 114.9, 67.6, 33.9, 29.9, 28.6, 6.9; MALDI-TOF-MS m/z: $C_{75}H_{110}Br_{10}O_{10}$ 1949.91 (found 1982.90, $[M+Na]^+$ and 1998.88, $[M+K]^+$).

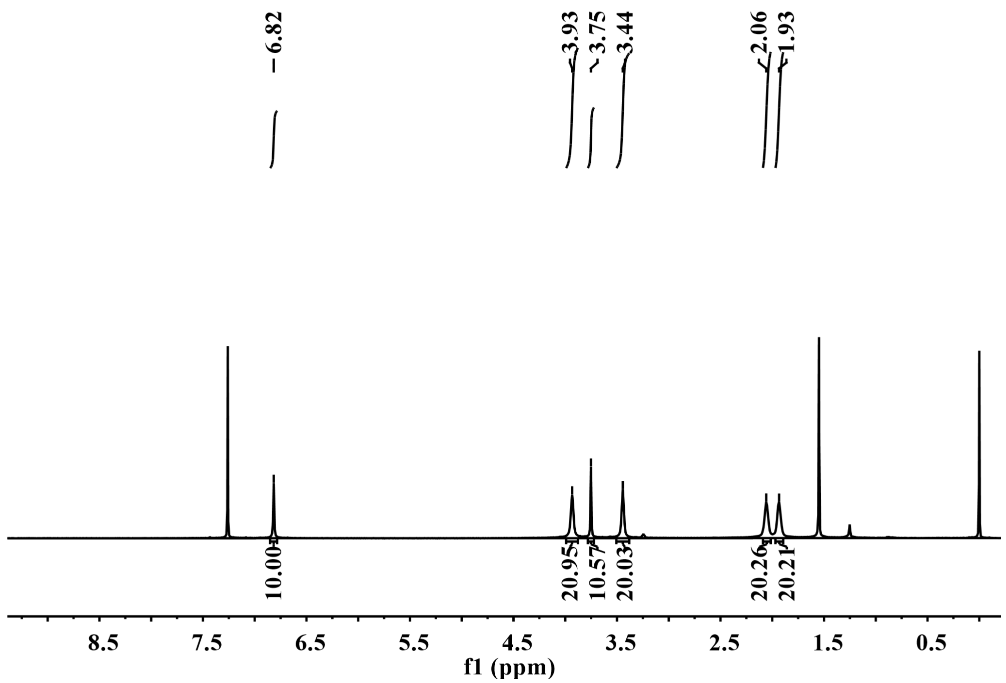

Figure S1 ^{1}H-NMR (600MHz, $CDCl_3$, 298K) spectra of compound **2**

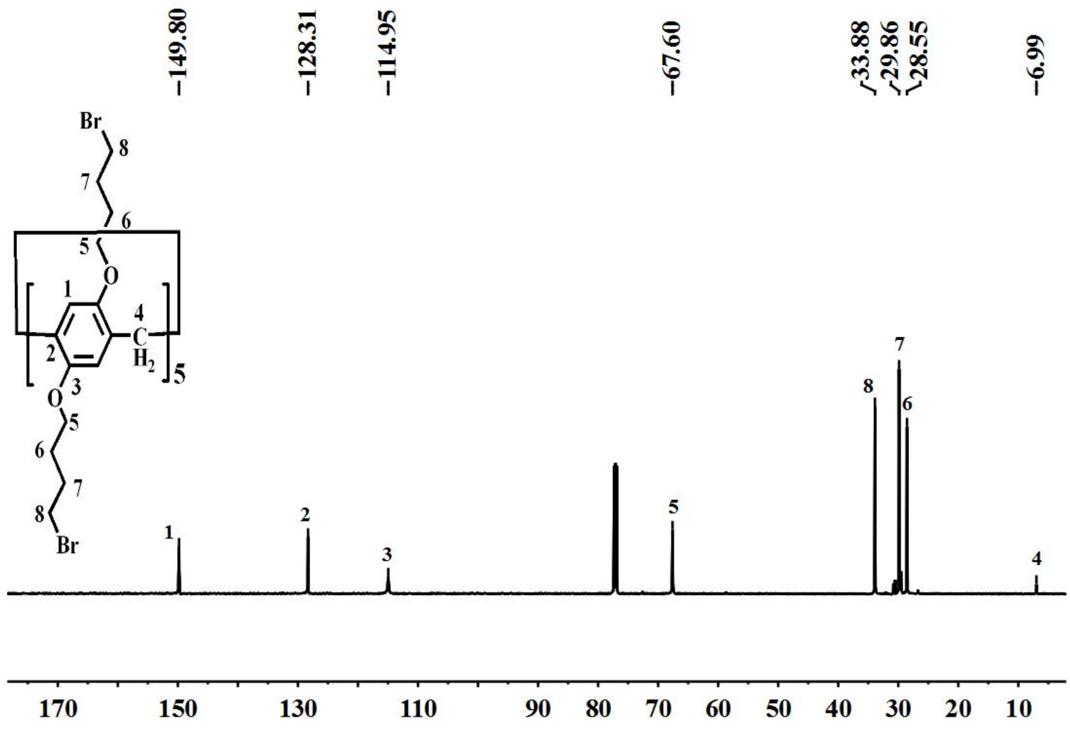

Figure S2 ^{13}C-NMR (151MHz, $CDCl_3$, 298K) spectra of compound **2**

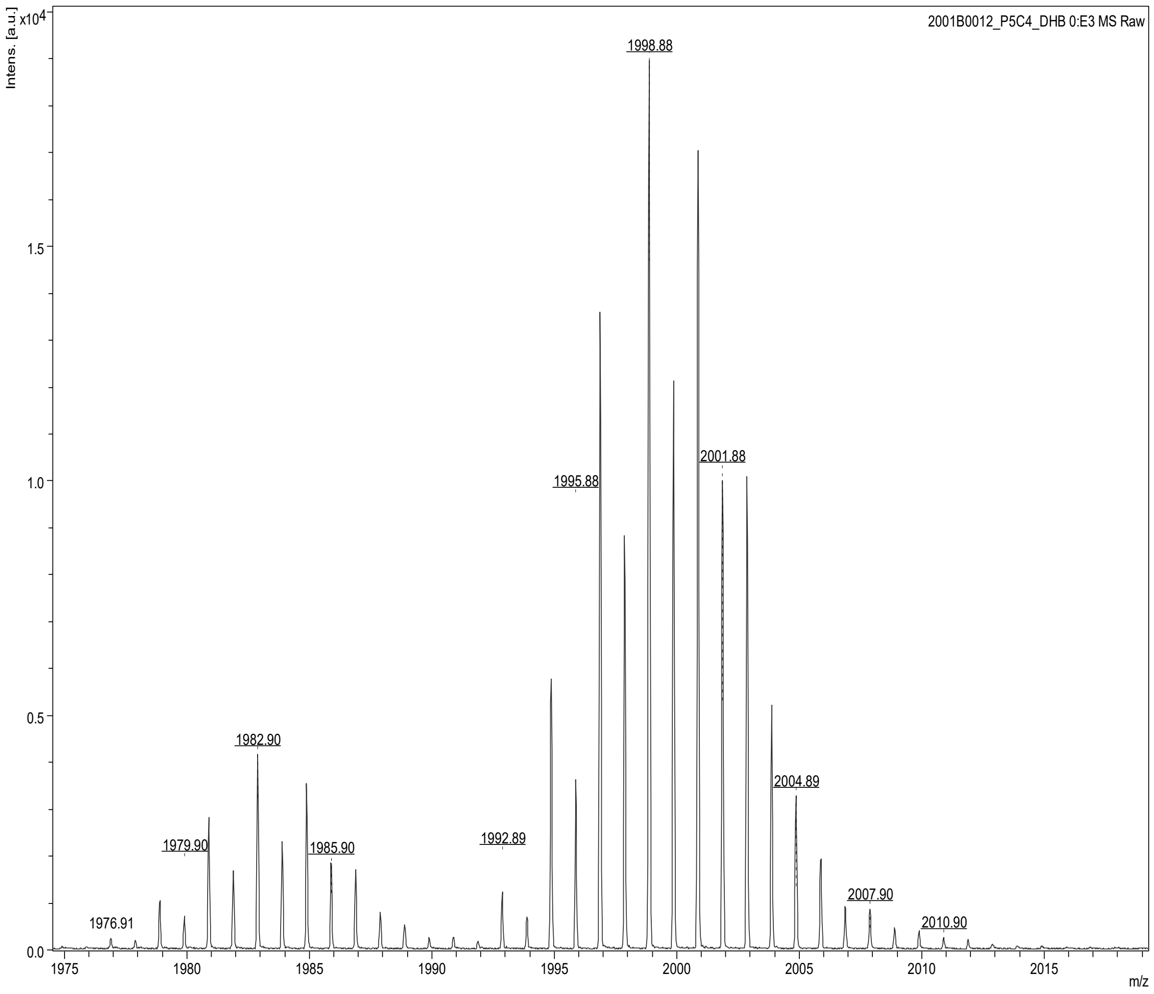

Figure S3 MALDI-TOF mass analysis of compound **2**

*Compound **3***

To a solution of compound **2** (1.2 g, 0.61 mmol) in acetonitrile (25 mL), alanine (2.2 g, 24.4 mmol) and potassium carbonate (3.37 g, 24.4 mmol) were added and the mixture was stirred for 6 h at 75 °C. Water (30 mL) was added to the residue, and then extracted with CH_2Cl_2 (3 ×30mL). The combined organic layer was dried over Na_2SO_4 and the dichloromethane was evaporated off. The crude product was purified by column chromatography using a mixture of ethyl acetate and petroleum ether giving compound **3** as a light yellow oil. Yield 90%; ^{1}H NMR (600 MHz, $CDCl_3$, 298K) δ/ppm: 6.79 (s, 1H), 5.35 (s, 1H), 4.24(m, 1H), 4.32 (m, 2H), 4.00 (d, J = 4.5 Hz, 1H), 3.79 (dd, J = 31.0, 22.7 Hz, 2H), 1.90 (dd, J = 18.4, 5.1 Hz, 4H), 1.46 (m, 9H), 1.39 (m, 3H); ^{13}C NMR (151 MHz, $CDCl_3$, 298K) δ/ppm: 173.47, 155.2, 149.75, 128.42, 115.13, 79.67, 67.87, 64.89, 49.24, 29.41, 28.34, 26.29, 25.68, 18.53; MALDI-TOF-MS m/z: $C_{155}H_{240}N_{10}O_{50}$ 3043.65 (found 3066.65, $[M+Na]^+$).

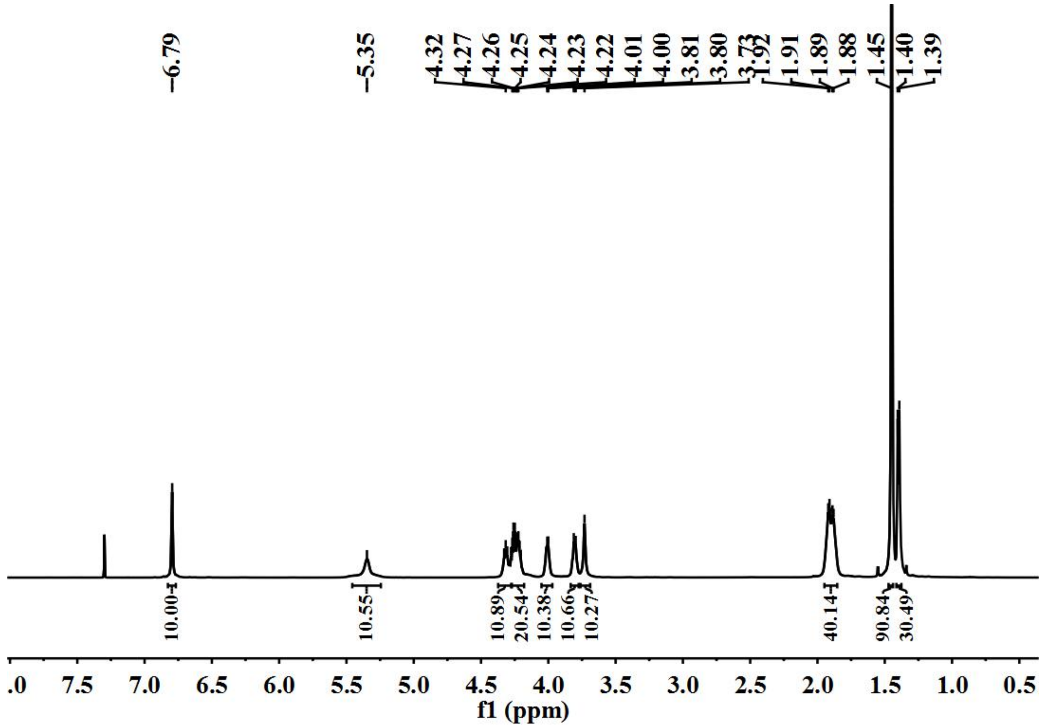

Figure S4 ^{1}H-NMR (600MHz, $CDCl_3$, 298K) spectra of compound **3**

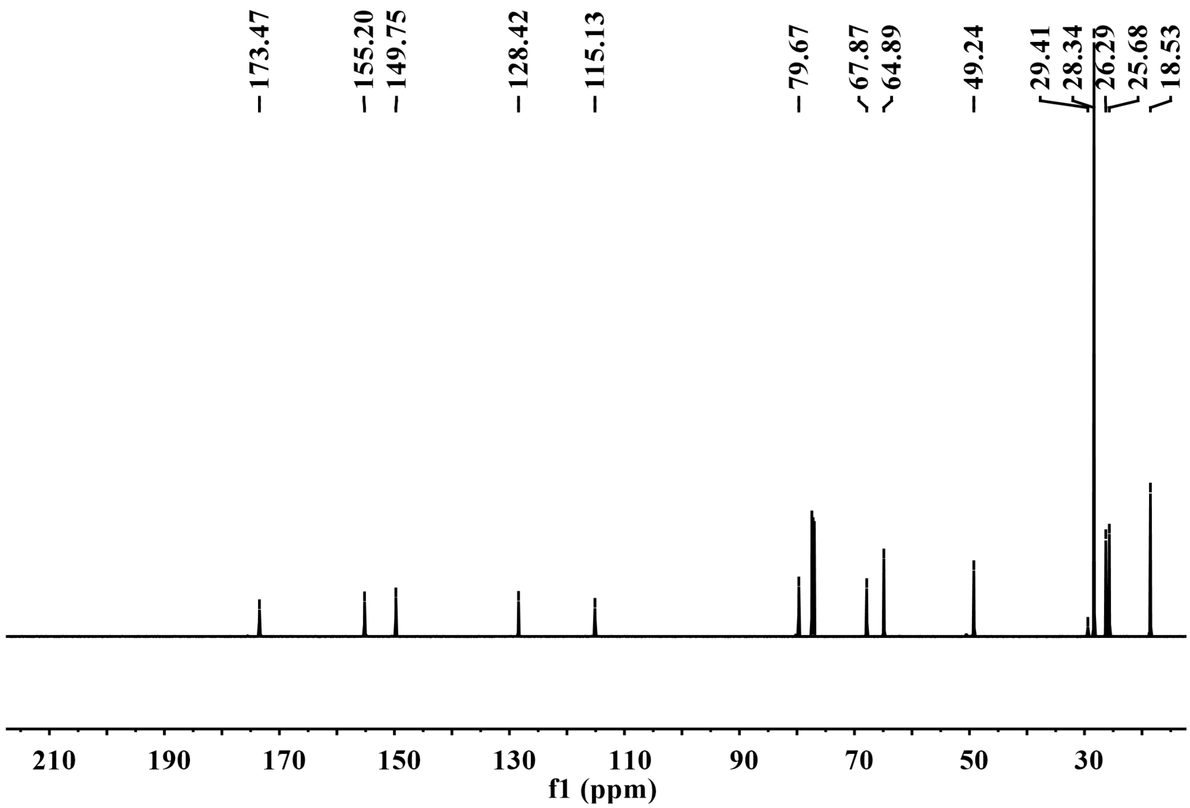

Figure S5 ^{13}C-NMR (151MHz, $CDCl_3$, 298K) spectra of compound **3**

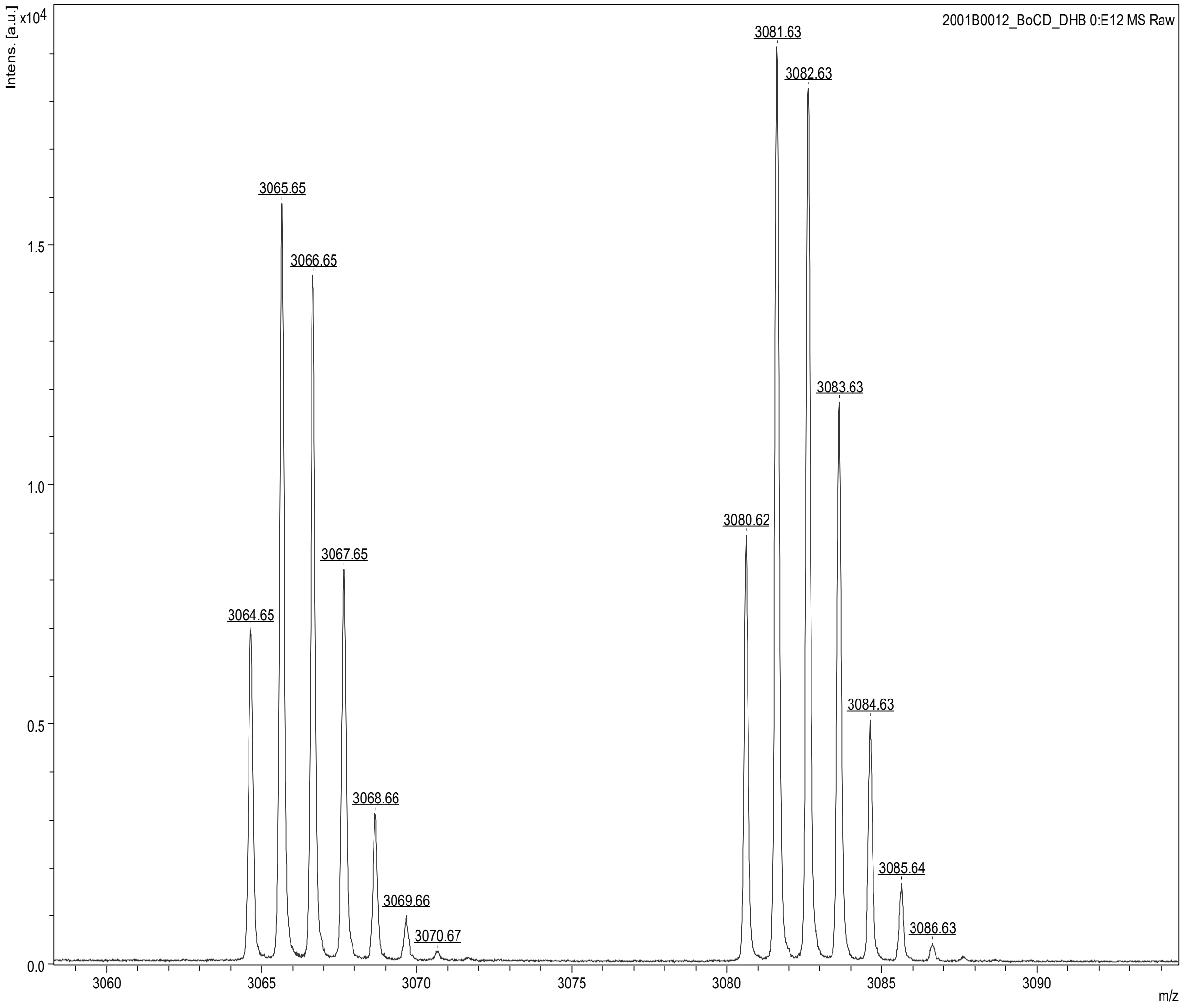

Figure S6 MALDI-TOF mass analysis of compound **3**

*Compound **DAWP5***

To a solution of compound **3** (0.6 g, 0.2 mmol) in 1, 4-dioxyclohexane (10 mL), hydrochloric acid gas was added and the mixture was heated to reflux and stirred for 6 h. The precipitate was filtered, dried and recrystallized from methanol/diethyl ether to give DAWP5 as yellow crystals. Yield 65%; mp 241.7 ºC, ^{1}HNMR (600 MHz, D_2O) δ/ppm: 6.73 (d, J = 39.9 Hz, 1H), 4.38 – 4.18 (m, 2H), 4.11 (dd, J = 12.9, 5.9 Hz, 1H)， 3.82 – 3.61 (m, 3H), 1.73 (d, J = 48.8 Hz, 4H), 1.52–1.39 (m, 3H); ^{13}C NMR (151 MHz, D_2O) δ/ppm: 170.79, 150.19, 129.30, 116.45, 69.11, 66.75, 48.80, 25.37, 24.90, 15.27, 14.1; MALDI-TOF-MS m/z: $C_{105}H_{160}N_{10}O_{30}$ 2042.13 (found 2042.12, $[M]^{+}$).

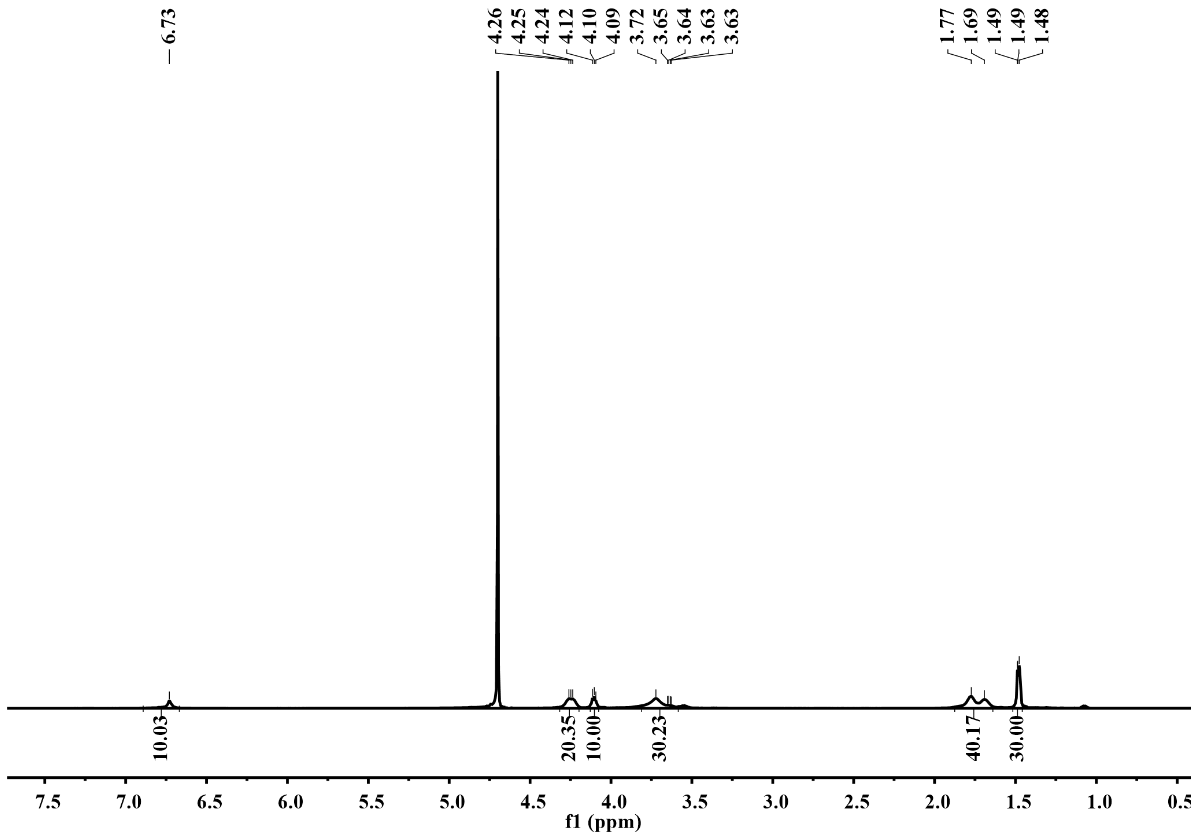

Figure S7 ^{1}H-NMR (600MHz, D_2O, 298K) spectra of compound **DAWP5**

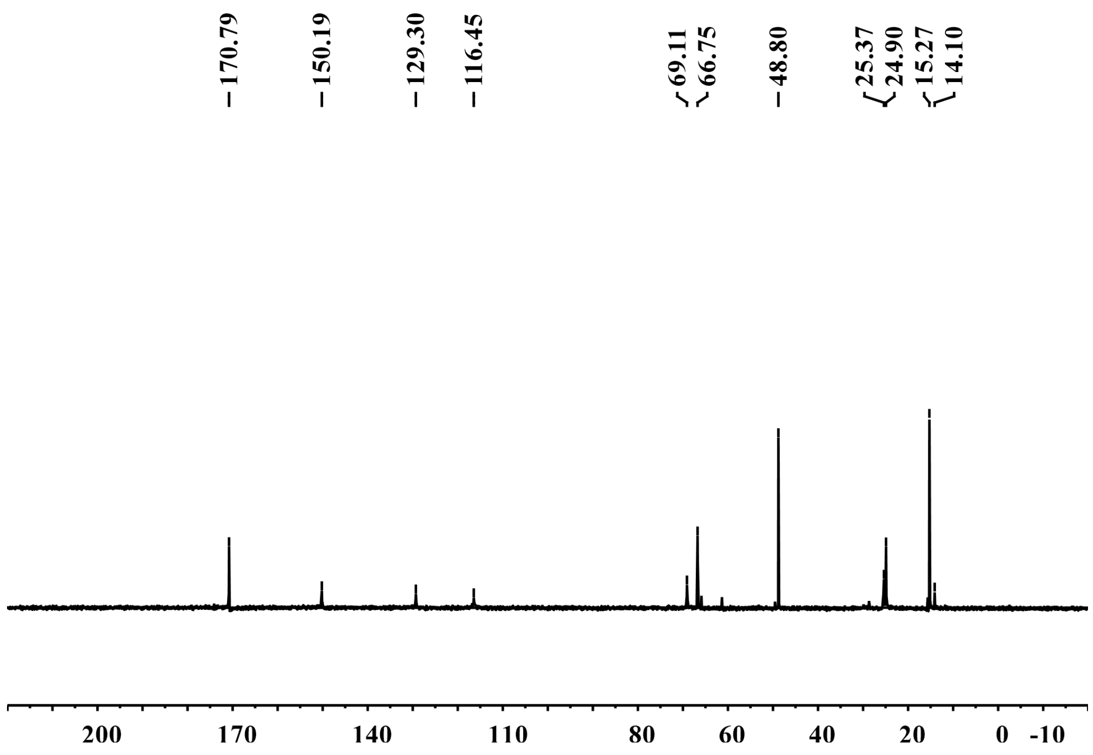

Figure S8 ^{13}C-NMR (151MHz, D_2O, 298K) spectra of compound **DAWP5**

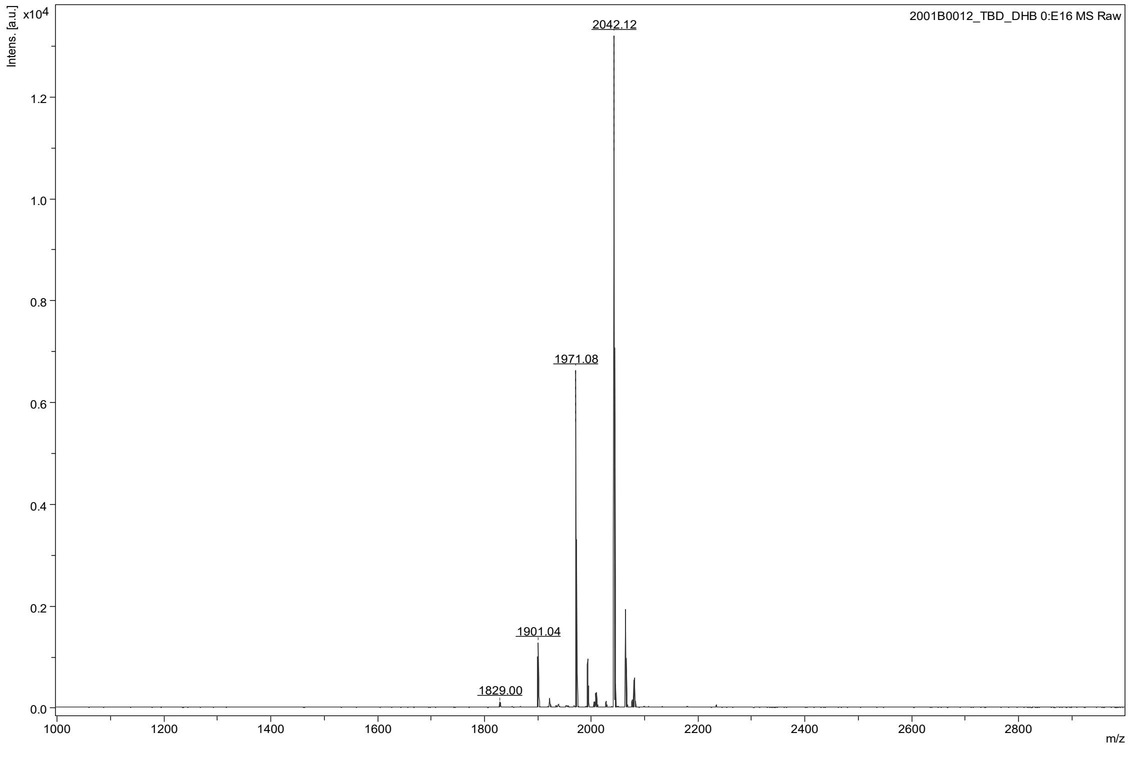

Figure S9 MALDI-TOF mass analysis of compound **DAWP5**

2 Job plot of complex SDS@DAWP5 and its association constant (Ka)

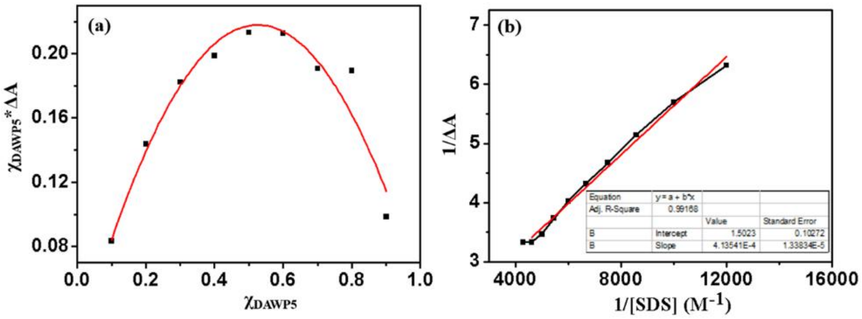

Figure S10 (a)Job plot showing the 1:1 stoichiometry of the complex between DAWP5 and SDBS in aqueous solution by plotting the absorbance difference at 295 nm (a characteristic absorption peak of DAWP5) against the mole fraction of DAWP5. (b) The absorbance changes of DAWP5 upon addition of SDS. The association constant of host-guest complex between DAWP5 and SDBS was calculated to be about $(3.5\pm0.1)\times10^{3}$ M^{-1} by a linear curve-fitting method based on the Benesi-Hildebrand equation.

$$\frac{1}{\Delta A} = \frac{1}{\Delta A_{0} K[G]} + \frac{1}{\Delta A_{0}}$$

3 Tyndall Effects of DAWP5 and SDS⊂DAWP5 Vesicles in Different pH

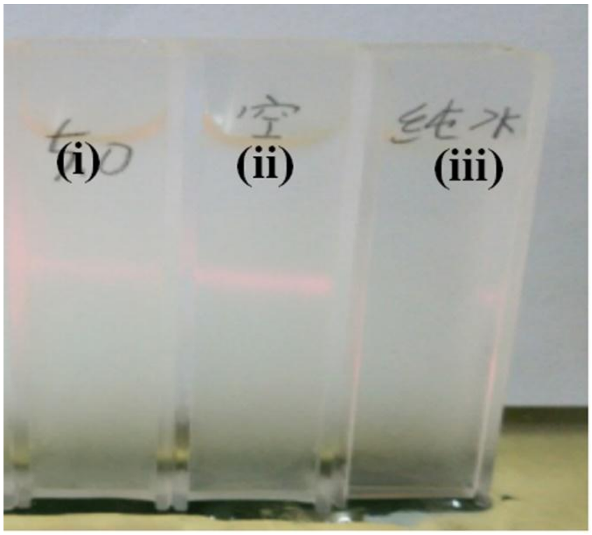

Figure S11 Photo showing the Tyndall effect of SDS⊂DAWP5 (i: pH=4.8, ii:pH=7.0) and DAWP5 (iii:pH=7.0) in aqueous solution

4 Determination of the Best Molar Ratio of DAWP5 and SDS for Aggregation

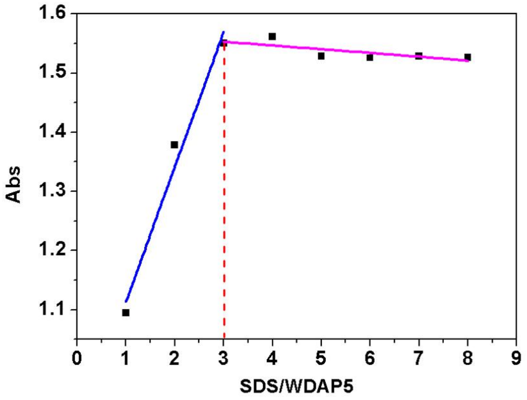

Figure S12 The absorbance of DAWP5 (100 µM) at 295 nm upon addition of SDS.

5 The Wall Thickness of Vesicles SDS⊂DAWP5 and the Length of SDS

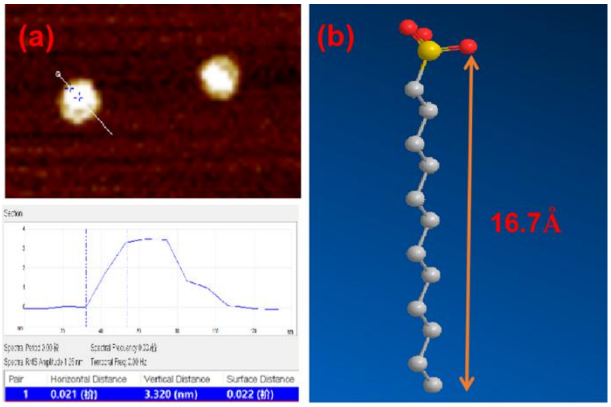

Figure S13 (a) The wall thickness of vesicles SDS⊂DAWP5. (b) The length of SDS is calculated by MM2 method.

6 DLS data of SDS⊂DAWP5 Vesicles (SDS/ADWP5=5/1) in Different pH

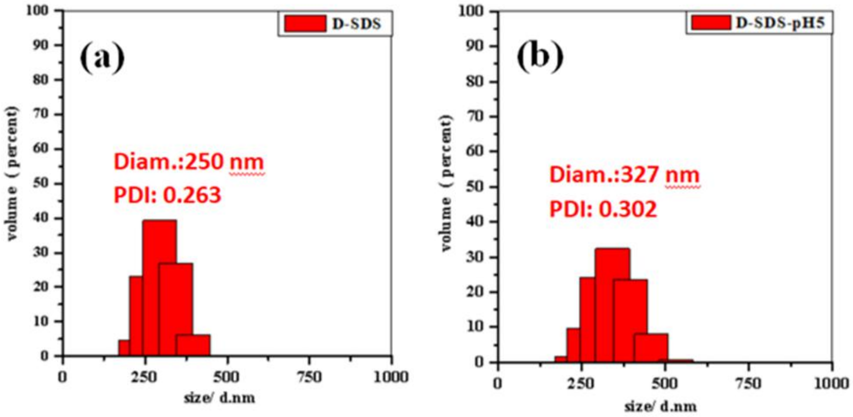

Figure S14 DLS data of SDS⊂DAWP5 vesicles (SDS/ADWP5=5/1) (a) pH=7.0; (b) pH=5.0.

7 SEM of SDS⊂DAWP5 Vesicles (SDS/ADWP5=5/1)

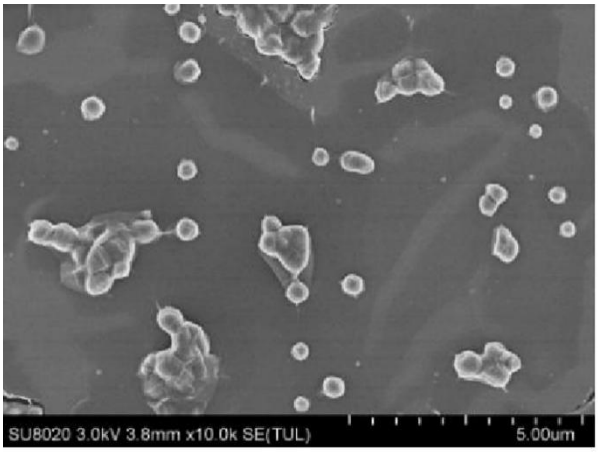

Figure S15 SEM images of SDS⊂DAWP5 (5/1) vesicles.
